# The complete genome of *Zunongwangia profunda *SM-A87 reveals its adaptation to the deep-sea environment and ecological role in sedimentary organic nitrogen degradation

**DOI:** 10.1186/1471-2164-11-247

**Published:** 2010-04-17

**Authors:** Qi-Long Qin, Xi-Ying Zhang, Xu-Min Wang, Gui-Ming Liu, Xiu-Lan Chen, Bin-Bin Xie, Hong-Yue Dang, Bai-Cheng Zhou, Jun Yu, Yu-Zhong Zhang

**Affiliations:** 1 State Key Lab of Microbial Technology, Marine Biotechnology Research Center, Shandong University, Jinan 250100, PR China; 2 CAS Key Laboratory of Genome Sciences and Information, Beijing Institute of Genomics, Chinese Academy of Sciences, Beijing, 100029, PR China; 3 Centre for Bioengineering and Biotechnology, China University of Petroleum (East China), Qingdao 266555, PR China

## Abstract

**Background:**

*Zunongwangia profunda *SM-A87, which was isolated from deep-sea sediment, is an aerobic, gram-negative bacterium that represents a new genus of *Flavobacteriaceae*. This is the first sequenced genome of a deep-sea bacterium from the phylum *Bacteroidetes*.

**Results:**

The *Z. profunda *SM-A87 genome has a single 5 128 187-bp circular chromosome with no extrachromosomal elements and harbors 4 653 predicted protein-coding genes. SM-A87 produces a large amount of capsular polysaccharides and possesses two polysaccharide biosynthesis gene clusters. It has a total of 130 peptidases, 61 of which have signal peptides. In addition to extracellular peptidases, SM-A87 also has various extracellular enzymes for carbohydrate, lipid and DNA degradation. These extracellular enzymes suggest that the bacterium is able to hydrolyze organic materials in the sediment, especially carbohydrates and proteinaceous organic nitrogen. There are two clustered regularly interspaced short palindromic repeats in the genome, but their spacers do not match any sequences in the public sequence databases. SM-A87 is a moderate halophile. Our protein isoelectric point analysis indicates that extracellular proteins have lower predicted isoelectric points than intracellular proteins. SM-A87 accumulates organic osmolytes in the cell, so its extracelluar proteins are more halophilic than its intracellular proteins.

**Conclusion:**

Here, we present the first complete genome of a deep-sea sedimentary bacterium from the phylum *Bacteroidetes*. The genome analysis shows that SM-A87 has some common features of deep-sea bacteria, as well as an important capacity to hydrolyze sedimentary organic nitrogen.

## Background

The average depth of the oceans is about 3 800 m, and almost 60% of the earth's surface is deep-sea floor (water depth greater than 2 000 m) [1]. Although most of the deep-sea floor environment is characterized by darkness, high hydrostatic pressure and low temperatures, more than half of the world's prokaryotes live in sub-seafloor sediments [2-4], which play a major role in marine biogeochemical cycling [5]. Every year, massive amounts of particulate organic matter (POM) are transported to the deep layers of the ocean floor, forming a large pool of carbon and nitrogen [6]. However, how this organic material is degraded, as well as the types of bacteria involved and the enzymes used, are still unclear, especially for the degradation of sedimentary organic nitrogen (SON) [7]. To date, numerous efforts have been made to clarify the mechanism of SON degradation, including investigations of the diversity of bacteria and proteases involved in SON degradation [8,9]. Researchers have also characterized some extracellular proteases from sedimentary bacteria and elucidated their ecological role in SON degradation [10-13]. However, the role of deep-sea bacteria in SON degradation and cycling has never been analyzed at a genomic level. Genome analyses of deep-sea heterotrophic bacteria would provide a better understanding of the deep-sea nitrogen cycle, and reveal to what extent bacteria affect the deep-sea environment [14].

*Bacteroidetes *(formerly *Cytophaga-Flavobacterium-Bacteroides *(CFB)) are a widespread and diverse group of bacteria that can be found throughout the sea, from surface water to deep-sea sediment. Studies of both cultivated and uncultivated marine *Bacteroidetes *have shown that *Bacteroidetes *are able to efficiently consume biopolymers such as protein and chitin [15,16], which make up a significant fraction of the high-molecular-weight dissolved organic matter (HMW DOM) pool in the ocean [17]. Biopolymer degradation is considered to be the rate-limiting step in DOM mineralization by marine microorganisms, and *Bacteroidetes *are hypothesized to play a key role in this process in the oceans [7]. Genome sequence data have been extremely helpful in the development of detailed hypotheses on the role of specific *Bacteroidetes *members in marine biogeochemical cycling. An analysis of the genome of *Bacteroidetes *'*Gramella forsetii*' KT0803, a bacterioplankton isolated from North Sea surface waters during a phytoplankton bloom, indicated that it is efficient at degrading biopolymers, especially proteins [18,19]. Metagenomic studies have also reported the distribution and functional analysis of specific *Cytophaga*-like hydrolases in the Sargasso Sea and western Arctic Ocean and described hydrolase-containing genome fragments of Antarctic marine *Bacteroidete*s [20,21]. However, while *Bacteroidetes *have been frequently encountered in the analysis of sedimentary bacterial diversity, no complete genome analysis of a deep-sea sedimentary *Bacteroidetes *has yet been published [15,16]. This type of analysis could be used to address how the organisms' genetic inventories reflect both their SON remineralization capabilities and their adaptation to the deep-sea environment.

*Wangia profunda *SM-A87 (hereafter called SM-A87), isolated at a depth of 1 245 m from deep-sea sediment in the southern Okinawa Trough with *in situ *temperature of 4.7°C, is a newly described species of *Bacteroidetes *and represents a new genus of *Flavobacteriaceae *[22]. It was renamed *Zunongwangia profunda *in the *International Journal of Systematic and Evolutionary Microbiology *(IJSEM) Validation List no. 116. In this study, we report its complete genome sequence, which represents the first genome of a deep-sea bacterium of the phylum *Bacteroidetes*. In addition, we performed a genomic comparison with two bacteria of the family of *Flavobacteriaceae *from surface seawater, and two bacteria from a cold deep-sea environment. Our genomic analysis of strain SM-A87 indicates that it is capable of degrading biopolymer sources and sheds light on its adaptation to the deep-sea environment.

## Results and discussion

### General genome features

General features of the *Z. profunda *SM-A87 genome are summarized in Table 1. The genome has a single 5.1-Mbp circular chromosome with no extrachromosomal elements. The G+C content of the genome is 36.2%, which is slightly higher than the experimentally determined 35.8% [22]. The genome harbors 4 653 predicted open reading frames (ORFs), of which 69.4% are annotated with known or predicted functions. About 50% of SM-A87 ORFs have the highest similarity to those in the published genome of *G. forsetii *KT0803. SM-A87 has 47 tRNA genes and three 16S-23S-5S operons.

**Table 1:** General features of the *Z. profunda *SM-A87 genome.

Size (bp)	5 128 187
G + C content	36.2%
Number of predicted ORFs	4 653
Average ORF length (bp)	960
Coding density	87.4%
tRNAs	47
rRNA operons	3
Conserved hypothetical proteins %	17.1%
Hypothetical proteins %	13.5%

**Figure 1:**
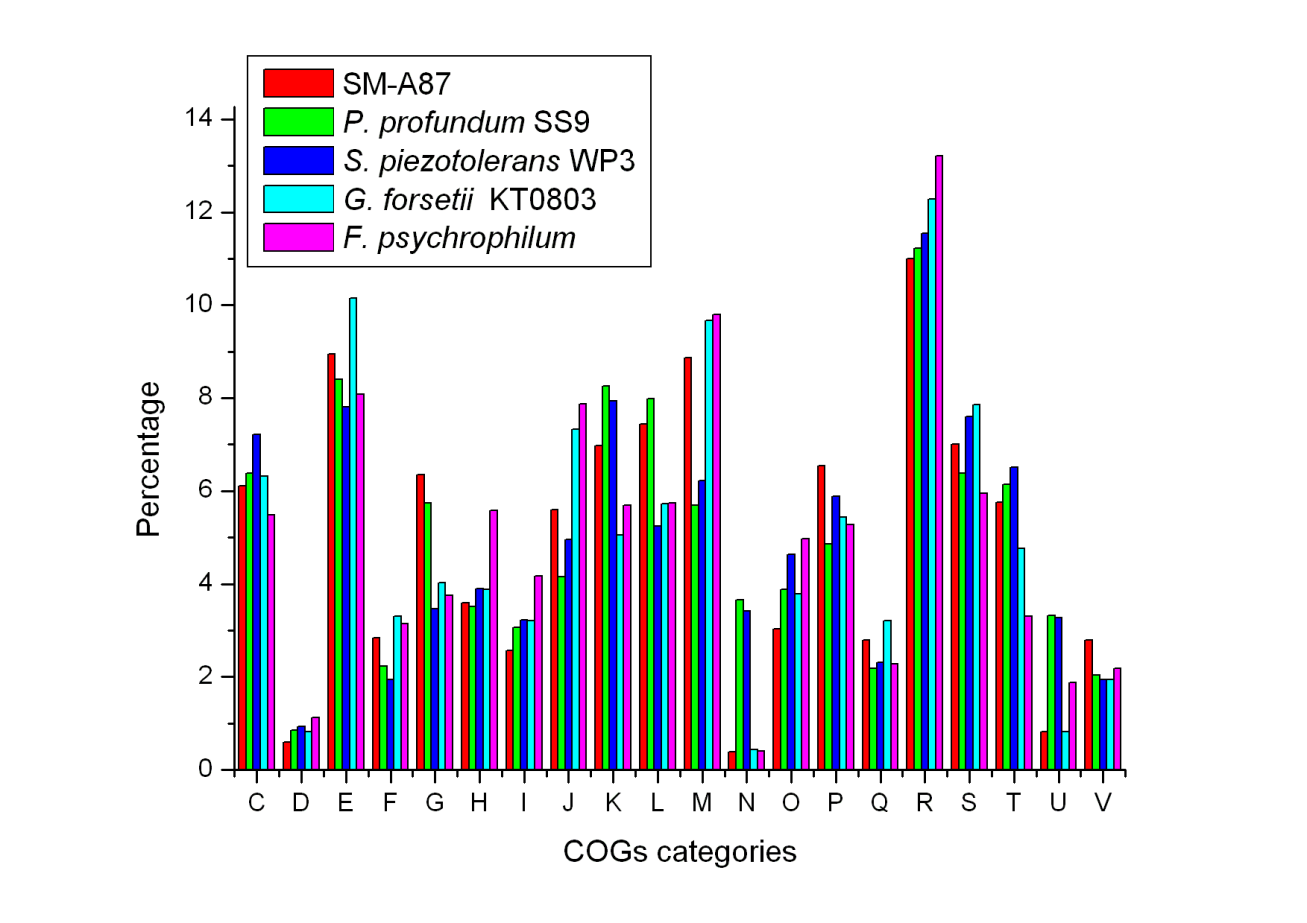
**COG category percentage of *Z. profunda *SM-A87 and other compared bacteria**.

Figure 1 shows the proportions of proteins belonging to clusters of orthologous groups (COGs) in SM-A87 and several other bacterial groups. The deep-sea bacteria (SM-A87, *Photobacterium profundum *SS9 and *Shewanella piezotolerans *WP3) have an average of 2.35% more proteins belonging to COG K (transcription), and 2.11% more proteins belonging to COG T (signal transduction mechanisms) than the shallow-water bacteria (*Flavobacterium psychrophilum *and *Gramella forsetii *KT0803). These differences are statistically significant (p < 0.05).

### General metabolism

SM-A87 has a full set of genes for glycolysis, the pentose phosphate pathway and the tricarboxylic/citric acid cycle. The strain has five predicted lactate dehydrogenases, two L- and three D-, and also contains predicted ethanol-producing enzymes including aldehyde dehydrogenase (ZPR_1384, ZPR_3649) and alcohol dehydrogenase (ZPR_4362). These enzymes may help the bacterium to survive in the low-oxygen environment of the deep sea. SM-A87 contains predicted cytochrome bd ubiquinol oxidase subunits I (ZPR_1985) and II (ZPR_1985). This oxidase is related to adaptation to microaerobic conditions in the deep sea [23]. According to the Kyoto Encyclopedia of Genes and Genomes (KEGG) pathway map of SM-A87, fructose and mannose can be converted to fructose-6p, and galactose can be converted to glucose-1p; both fructose-6p and glucose-1p are then degraded through glycolysis. SM-A87 harbors a predicted keto-deoxy-phosphogluconate aldolase (ZPR_2957), which is the key enzyme of the Entner-Doudoroff metabolic pathway. This strain also contains the enzymes that utilize most amino acids. All these features confer a metabolic versatility to SM-A87 that allows it to utilize sparse and sporadic nutrients in the deep-sea environment.

SM-A87 has all the genes required for fatty acid oxidation. However, 3R-hydroxymyristoyl ACP dehydrase, a component of the fatty acid biosynthesis pathway, was not found by genome annotation. This absence may be due to the low similarity of this gene to those in the databases or to convergent evolution from other functionally similar enzymes with divergent sequences. Phosphatidylethanolamine is the only phospholipid that has been experimentally identified in *Zunongwangia profunda *[22]. The genome analysis suggests that phosphatidylethanolamine is derived from phosphatidylserine, which is synthesized from glycerate.

SM-A87 has all of the components of the oxidative phosphorylation pathway but does not contain any rhodopsin or retinal genes, consistent with the dark deep-sea environment in which the strain thrives.

### Sensing system

Most nutrients arrive in the deep sea in an annual pulse, and the bacteria in the deep sea can sense this food pulse and respond accordingly [24]. A widespread sensing system used by bacteria is the two-component signal transduction system, which consists of a signal sensor histidine kinase and a response regulator [25,26]. SM-A87 harbors 47 predicted sensor histidine kinases, the most of any of the compared strains (Table 2), which indicates its strong ability to sense environment signals. SM-A87 has nine predicted two-component operons, each of which is composed of a histidine kinase and a response regulator that may form a one-to-one phosphotransfer pair. SM-A87 has three rRNA operons, suggesting that it can respond to nutrient enrichment rapidly and grow quickly [27]. SM-A87 can form colonies of 1-3 mm in diameter on a rich medium after 48 h of cultivation at 28°C [22].

**Table 2:** Comparison of the numbers of selected proteins between SM-A87 and other four marine bacteria.

		SM-A87	*P. profundum* SS9	*S. piezotolerans* WP3	*G. forsetii* KT0803	*F. psychrophilum*
Sensing components	Histidine kinase	47	20	46	37	13
	RagB/susD family protein	22	0	0	14	0
Enzymes for degradation	Peptidase^1^	130 (60)	74 (18)	129 (50)	94 (47)	59 (42)
	Glucosidase	11	0	5	5	0
	Xylanase	6	0	0	0	0
	Xylosidase	3	0	0	1	0
	Beta-galactosidase	5	3	2	4	0
	Amylase	3	2	4	3	0
	Chitinase	2	3	2	0	0
	Other glycosidase	50	4	6	21	2
	Lipase	14	11	7	9	3
	Esterase	46	20	32	22	7
Transportors	ABC-type transporter	40	155	64	28	40
	TonB-dependent receptor	40	0	33	40	22

^1^In parentheses is the number of proteins with signal peptides.

SM-A87 contains 22 genes encoding RagB/SusD family proteins in its genome, while the other compared genomes have fewer or none (Table 2). RagB is a protein involved in signaling and SusD is an outer membrane protein involved in nutrient binding [28,29]. Nineteen of the RagB/SusD family protein genes are each adjacent to a TonB-dependent receptor gene, forming 19 predicted operons. Of the 19 predicted operons, 12 are adjacent to predicted glycosyl hydrolases or peptidases [see Additional file 1]. Specifically for the genes from ZPR_1020 to ZPR_1033, there are two glycosidase genes, two esterase genes, one xylanase gene and seven glycosyl hydrolase genes next to the operon, implying that SM-A87 can sense and respond to sugar sources. The genome also contains 27 putative outer membrane protein genes, which are probably involved in nutrient binding. All these features imply that SM-A87 has the ability to sense extracellular nutrients such as sugars and proteins.

### Polysaccharide synthesis

Many deep-sea bacteria produce exopolysaccharides that help them survive in the extreme deep-sea environment [30]. Reports suggest that these polysaccharides help the bacteria concentrate organic matter, absorb metal ions, and form biofilms in the marine environment [31,32]. Different glycosyltransferases can contribute to the biosynthesis of disaccharides, oligosaccharides, and polysaccharides [33]. Our experimental results show that strain SM-A87 can produce large quantities of capsular polysaccharide (data not shown). The genome analysis shows that SM-A87 contains 46 predicted glycosyl transferases, of which 13 belong to family two and 10 belong to family one. SM-A87 contains two glycosyl transferases (ZPR_0565 and ZPR_1126) that are similar to WbaP from *Salmonella enterica*. WbaP is responsible for the initiation of polysaccharide synthesis, transferring the first sugar to undecaprenyl phosphate (Und-P) [34]. Similar to WbaP, ZPR_1126 has 462 amino acid residues and five predicted transmembrane regions. The topological organization of the transmembrane regions of these two enzymes is similar: there are four transmembrane regions at the N-terminus and one transmembrane domain with sugar-phosphate transferase activity at the C-terminus. ZPR_0565 consists of 339 amino acid residues and has only one transmembrane domain. A multiple sequence alignment [see Additional file 2] shows that ZPR_0565 corresponds to the C-terminus of WbaP and other initial glycosyltransferases; in addition, they all contain the highly conserved amino acid motifs KFRSM, DELPQ, and PGITG [35]. This implies that ZPR_0565 contains only the glycosyltransferase catalytic domain. Genes encoding other glycosyltransferases and polysaccharide export proteins are found close to ZPR_0565 and ZPR_1126; together, these genes form two gene clusters for polysaccharide synthesis and export [see Additional file 3]. ZPR_1123, which is upstream of ZPR_1126, is predicted to encode an O-antigen polymerase, implying that polysaccharides are synthesized through the Wzy-dependent pathway in SM-A87 [36]. SM-A87 harbors two predicted capsular polysaccharide biosynthesis proteins that are probably involved in the synthesis of capsular polysaccharides. The production of capsular polysaccharide is advantageous for SM-A87 to thrive in the marine environment.

### Hydrolysis ability

Signal peptide analysis suggests that SM-A87 can secrete a large number of hydrolysis enzymes, and it has more exported peptidases than the other compared strains (Table 2), reflecting its unusual ability to degrade organic nitrogen. SM-A87 contains 130 predicted peptidases, 61 of which have signal peptides. The peptidases with signal peptides have more aspartic acids and a higher ratio of acidic residues to basic residues. Additionally, they have a lower predicted isoelectric point (pI) than the peptidases without signal peptides (Table 3), a difference that is statistically significant (p < 0.05). Thus, the extracellular peptidases are more halophilic than the intracellular peptidases, as high numbers of acidic residues and low pIs are key features of halophilic proteins [37]. The halophilicity of the extracellular peptidases helps them function in saline environments and decompose extracellular organic nitrogen matter in the marine salty condition.

**Table 3:** Properties of peptidases with and without signal peptides.

	With signal peptides	Without signal peptides
Number	61	69
G+C content (%)	37.5	36.8
Asp (percentage)	6.6	6.0
Glu (percentage)	7.2	7.2
Lys (percentage)	8.0	8.1
Arg (percentage)	3.6	3.5
(Asp+Glu)/(Lys+Arg)	1.2	1.1
pI^1^	6.0 ± 1.7	6.7 ± 2.0

^1^pI, predicted isoelectric point. The data are averages with standard deviation.

**Figure 2:**
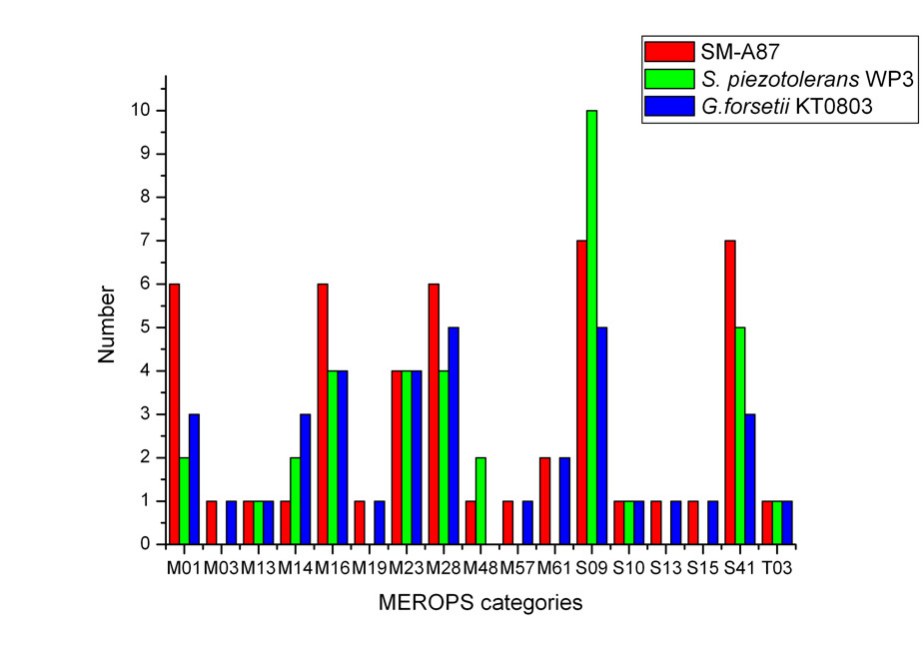
**The MEROPS category of the extracellular peptidases from *Z. profunda *SM-A87, *G. forsetii *KT0803 and *S. piezotolerans *WP3**.

Fifty-two of the SM-A87 peptidases with signal peptides can be assigned to different families in the MEROPS peptidase database [38]. As shown in Figure 2, the peptidases mainly belong to families of metallopeptidases and serine peptidases, consistent with our previous study that the extracellular peptidases of marine sedimentary bacteria are mainly serine proteases and metalloproteases [9]. Compared to *G. forsetii *KT0803, deep-sea bacteria SM-A87 and *S. piezotolerans *WP3 have more peptidases in families S09 and S41, but fewer in family M14 (Figure 2). The variety of extracellular peptidases suggests that SM-A87 has the capacity to decompose diverse peptides and proteins from its surroundings. For instance, SM-A87 has six exported family M01 peptidases, which are aminopeptidases. It has been reported that when marine bacteria grow on HMW dissolved organic nitrogen (DON) as the sole nitrogen source, aminopeptidase activity is greatly enhanced [39]. Aminopeptidase activity in the deep-sea sediment is higher than that in the surface seawater; however, this is not the case for other hydrolysis enzymes [40]. The large number of aminopeptidases secreted by SM-A87 may contribute to the high aminopeptidase activity in the deep-sea sediment, and suggests that SM-A87 may be able to respond to HMW DON and decompose it. SM-A87 secretes seven S09 peptidases, which are prolyl oligopeptidases that cannot degrade peptides of more than 30 residues in length [41]. Therefore, the S09 peptidases specifically hydrolyze oligopeptides. Notably, there are six exported peptidases that contain a PDZ domain, which is known to be involved in peptide binding [42]. The large number of PDZ domain-containing peptidases secreted by SM-A87 suggests a strategy for binding and degrading proteins, similar to the PKD domains of exported proteins in *G. forsetii *KT0803 [19]. In the deep-sea sedimentary nitrogen cycle, the process by which particulate organic nitrogen is converted to NH_4_^+ ^is known to be dominated by bacteria but is poorly characterized [1,14]. The secreted peptidases of SM-A87 may play an important role in this process.

Among the compared strains, SM-A87 has the largest proportion of proteins belonging to carbohydrate transport and metabolism COGs (Figure 1). Accordingly, it has many genes encoding enzymes that degrade oligo- and polysaccharides (Table 2) [Additional file 4]. SM-A87 has 50 annotated glycosidases, 17 of which have signal peptides, suggesting that SM-A87 can hydrolyze extracellular carbohydrates. It does not contain any predicted cellulases, agreeing with the experimental result that it can not hydrolyze cellulose. Although SM-A87 has a gene for exported chitinase (ZPR_1703), experiments suggest that it does not degrade chitin. SM-A87 harbors six xylanase genes and three xylosidase genes, of which three xylanases and two xylosidases have signal peptides, indicating that the strain should have the ability to degrade xylan. The genome also has four genes encoding exported beta-galactosidases; correspondingly, beta-galactosidase activity has been detected in this strain [22].

SM-A87 contains 11 predicted glucosidases, of which seven have signal peptides. Glucosidase production can be induced by dissolved polymeric glucose [43]. The large number of glucosidases in SM-A87 indicates that it is able to decompose the easily used polymeric sugar in the environment. The carbohydrate-hydrolyzing enzymes mentioned above imply that SM-A87 is able to use a variety of carbohydrates in the environment as carbon and energy sources.

SM-A87 harbors seven genes encoding extracellular lipases, including one phospholipase A1 (ZPR_0295) and two GDSL family lipases. The strain contains 20 genes encoding esterases with signal peptides, of which five are carboxyl esterases and six are phosphoesterases. These enzymes may allow SM-A87 to degrade various phospholipids and carboxyl esters in the environment as carbon and phosphorus sources.

DNA is abundant in the deep-sea sediment, and most of it is extracellular. More than half of the total extracellular DNA can be rapidly degraded by enzymes [44]. DNA can be utilized by bacteria as a source of carbon, nitrogen, and phosphorous, contributing to phosphate recycling [44]. SM-A87 harbors two predicted extracellular endonucleases (ZPR_0199, ZPR_1186), implying that it can obtain nutrient elements by degrading extracellular DNA.

It has been reported that sedimentary carbohydrate is hydrolyzed more easily when it is treated with four enzymes, α-amylase, β-glucosidase, protease and lipase, than when it is treated with only one enzyme [45]. SM-A87 contains these four kinds of extracellular enzymes, implying that the strain can hydrolyze sedimentary carbohydrates easily. The variety of exported peptidases and other hydrolysis enzymes in the SM-A87 genome would allow the bacterium to degrade sedimentary biopolymeric materials into small molecules that can be absorbed by the cell. A previous genome analysis revealed that *G. forsetii *KT0803 from surface seawater is good at degrading polymeric organic materials [19]. Our analysis of the SM-A87 genome indicates that this deep-sea sediment *Bacteroidetes *species also has the unusual ability to decompose polymeric organic materials, which could contribute considerably to deep-sea sedimentary biogeochemistry cycles.

### Nitrogen and sulfur metabolism

According to the KEGG pathway map, nitrite reductase can catalyze the conversion of nitrite to ammonia [46]. Two nitrite reductases (ZPR_3631, ZPR_4195) could be responsible for the conversion of nitrite to ammonia in SM-A87. One formate/nitrite transporter (ZPR_2292) and three putative nitrate/nitrite DNA-binding response regulators in the genome indicate that SM-A87 can absorb nitrite from the environment. All of the above evidence suggests that the strain can use inorganic nitrogen.

The SM-A87 genome encodes one sulfate transporter (ZPR_0777) and two sodium:sulfate symporters (ZPR_0364, ZPR_4168) that can transport sulfate ions into the cell. It also has corresponding enzymes to utilize sulfate. There are three adjacent ORFs that encode adenylylsulfate kinase (ZPR_0539) and two subunits of sulfate adenylyltransferase (ZPR_0540, ZPR_0541), which convert sulfate to adenylyl sulfate (APS) and subsequently to 3'-phosphoadenylyl sulfate (PAPS). Phosphoadenosine phosphosulfate reductase (ZPR_3632) can convert PAPS into sulfite, and then hydrogen sulfide (H_2_S) could be produced from sulfite by the sulfite reductases (ZPR_0424, ZPR_4048). However, our previous experiments showed that SM-A87 does not secrete H_2_S (22). Thus, H_2_S is probably used to produce acetate via cysteine synthase (ZPR_2076), which converts 3-O-acetyl-L-serine (from serine) and H_2_S to acetate.

### Substrate transport systems

Since SM-A87 has many extracellular hydrolytic enzymes, there must be related systems to transport the nutrient products into the cell. The ATP-binding cassette (ABC) transporters, which are widespread among bacteria, can couple ATP hydrolysis to the transport of a variety of substrates into and out of the cell [47]. An examination of SM-A87 genome identified many genes encoding possible ABC transporters (Table 2), which is consistent with the report that there is an enrichment of ABC transporter genes in the genomes of deep-sea microorganisms [48]. SM-A87 has three predicted amino acid permeases and three amino acid transporters, which make it possible for the strain to absorb amino acids and oligopeptides. According to the KEGG pathway map, the strain may be able to transport molybdate, iron complexes, lipopolysaccharides and lipoproteins. In addition, the presence of genes encoding xylose permease (ZPR_0446), fucose permease (ZPR_4359) and glucose/galactose transporter (ZPR_0518) may correspond to the carbohydrate transport ability of SM-A87.

The TonB-dependent transport system can take up large substrate molecules, such as siderophores and vitamins, into the cell from the environment [49]. The genome analysis showed that 40 TonB-dependent receptor genes and 5 TonB protein genes are present in SM-A87. Consistent with previous reports, there are fewer TonB proteins than TonB receptors [50]. The predicted TonB-dependent siderophore receptors (ZPR_0148, ZPR_2532) are likely to be involved in the enrichment and transport of iron into the cell.

### Mobile elements

SM-A87 is predicted to carry many transposases, supporting the idea that transposases are abundant in the deep-sea environment [5,48]. Two predicted putative transposons were identified in the SM-A87 genome. One is composed of ZPR_3981, ZPR_3982 and ZPR_3983, of which ZPR_3981 and ZPR_3983 belong to the IS66 family of transposases. Surprisingly, ZPR_3982 is predicted to be an RNA-directed DNA polymerase (reverse transcriptase), implying that the DNA segment of ZPR_3982 may have come from an RNA virus. The second transposon is composed of ZPR_1509, ZPR_1511 and ZPR_1512, of which ZPR_1509 is an IS3 family transposase and ZPR_1512 is a IS116/IS110/IS902 family transposase. ZPR_1511 encodes a glyoxalase family protein with the highest amino acid similarity (63%) to a glyoxalase from *Myxococcus xanthus*.

### CRISPR

Clustered regularly interspaced short palindromic repeat (CRISPR) elements are common in bacteria and archaea. A CRISPR is characterized by direct repeats (DR) that are separated by similarly sized non-repetitive spacers. There are also CRISPR-associated (CAS) genes and a leader sequence before the repeat area. CRISPR elements can be described as follows: CAS genes-leader-DR_1_-spacer_1_- DR_2_-spacer_2 _...DR_n-1_-spacer_n-1_-DR_n_, where n is the number of repeats [51,52]. A single CRISPR locus has been detected in many bacterial genomes [52]. However, there are two predicted CRISPR loci in the SM-A87 genome. The first locus is 5 594 bp in length (bp 1 642 261 to bp 1 647 855). In the first locus, the DR sequence is 37 bp in length with 76 spacers. Six CAS genes of the cas1, cas2, cas3 and cas5 families were detected upstream of the first locus. The second locus is 2 114 bp in length (bp 4 768 893 to bp 4 771 007), with a 47-bp DR sequence and 26 spacers. There are only two CAS genes upstream of the second locus, one belonging to the cas1 family and one to the cas2 family. The two CAS systems are classified as different types according to the family and arrangement of the CAS genes.

CRISPR is thought to function as an anti-phage defense system via an RNA-silencing-like mechanism, and the spacers are often found to share high sequence similarities with phage sequences [52]. However, our BLAST searches resulted in no hits in the public databases, probably because only a small fraction of phage sequences are deposited in the databases. In the ocean, the quantity of phages is about 5-10 times more than that of bacteria [53]. SM-A87's CRISPRs may help defend against infection by unknown phages in the deep sea.

### Adaptation to salt and cold

Halophiles have two different ways to maintain their cellular osmotic balance in salty environments: by accumulating organic compatible solutes or by maintaining a high concentration of ions such as potassium in the cell [54,55]. SM-A87 is a moderate halophile and can tolerate 0-12% NaCl [22]. To analyze the mechanism of SM-A87's adaptation to the marine salty environment, we predicted and compared the pIs of intracellular and extracellular proteins from SM-A87, *G. forsetii *KT0803, hyperhalophilic bacterium *Salinibacter ruber*, nonhalophilic bacterium *Escherichia coli *and *Bacteroidetes *soil bacterium *Cytophaga hutchinsonii *(Table 4). Halophilic proteins contain more acidic residues and have lower pIs than nonhalophilic proteins [55]. *E. coli *is not halophilic, and there is no statistically significant difference between the pIs of its intracellular and extracellular proteins. *S. ruber *is a hyperhalophilic bacterium that has a high intracellular potassium concentration to maintain its cellular osmotic balance [55]; thus, the ion concentrations inside and outside of the cell are both high. The pIs of both the intracellular and extracellular proteins of *S. ruber *are much lower than those of *E. coli *and *C. hutchinsonii *(Table 4), suggesting that the *S. ruber *proteins are all halophilic. For the marine bacteria SM-A87 and *G. forsetii *KT0803, the intracellular protein pIs are higher than those of the extracellular proteins, with a statistically significant difference (p < 0.01 for all proteins, p < 0.05 for peptidases). This indicates that the intracellular proteins of SM-A87 have poor salt-tolerance, and it is therefore impossible for SM-A87 to maintain high ion concentrations in the cell for osmotic balance. Instead, SM-A87 has a glycine betaine transporter (ZPR_3842). Glycine betaine is a well-known osmoregulator, implying that SM-A87 may use organic compatible solutes rather than ions to maintain its cellular osmotic balance. Thus, the intracellular proteins of SM-A87 are not halophilic and have higher pIs. In contrast, the extracellular proteins of SM-A87 must face the moderately high ion concentration (~3%) of the sea, so these proteins are halophilic and have low pIs. This is also the case for *G. forsetii *KT0803, which also contains glycine betaine transporter. The salt-tolerance of the extracellular proteins of SM-A87 and their lower pIs are indicative of their adaptation to the salty marine environment.

**Table 4:** Predicted isoelectric points of the proteins of SM-A87 and compared strains^1^.

	SM-A87		*G. forsetii KT0803*		*E. coli*		*C. hutchinsonii*		*S. rubber*	
	**With signal** **peptides**	**Without signal** **peptides**	**With signal** **peptides**	**Without signal** **peptides**	**With signal** **peptides**	**Without signal** **peptides**	**With signal** **peptides**	**Without signal** **peptides**	**With signal** **peptides**	**Without signal** **peptides**
All proteins	6.29 ± 2.08	7.28 ± 2.21	5.73 ± 1.90	7.07 ± 2.20	7.22 ± 2.17	7.20 ± 2.11	7.45 ± 1.50	6.47 ± 1.42	6.58 ± 2.52	5.79 ± 2.15
Peptidases	5.96 ± 1.65	6.65 ± 2.02	5.29 ± 1.43	5.93 ± 1.66	7.54 ± 2.38	7.33 ± 2.13	7.65 ± 1.95	7.35 ± 1.93	5.76 ± 1.90	5.91 ± 2.17

^1 ^The data are averages with standard deviation.

Unsaturated fatty acids can increase the fluidity of the membrane, which is a common strategy used by bacteria to adapt to a cold environment. The membranes of deep-sea bacteria contain a high proportion of monounsaturated fatty acids, which are very important for maintaining bacterial membrane fluidity [56]. SM-A87 has five fatty acid desaturase genes, which may contribute to high membrane fluidity, and thus cold adaptation. In addition to the chaperones GroEL and DnaJ, SM-A87 has four cold shock protein genes and one heat shock protein gene, which may help the strain survive in the cold deep-sea environment. SM-A87 also has genes encoding the pyruvate dehydrogenase complex and trehalose phosphate synthase (ZPR_2459), which are both associated with cold adaptation [57].

## Conclusion

This work presents the first complete deep-sea bacterial genome of a member of the phylum *Bacteroidetes*. SM-A87 has some features that are common in deep-sea bacteria, such as numerous transposases and ABC-type transporters. Our genome survey also reveals its metabolic versatility and extensive hydrolytic capabilities.

Additionally, based on the contents of its genome, SM-A87 can sense nutrient pulses, synthesize exopolysaccharides to absorb nutrients, export a variety of enzymes to degrade materials, transport substrates into the cell efficiently and utilize resources via versatile metabolic pathways. With these features, SM-A87 can thrive in the deep-sea environment.

## Methods

Strain SM-A87, originally isolated from deep-sea sediment in the southern Okinawa Trough, was cultured as previously described [22]. The cells were harvested by centrifugation at 12 000 g at 10°C for 30 min. Genomic DNA was prepared by using a genomic DNA extraction kit (BioTeke, China) according to the manufacturer's instructions.

The genome sequence of strain SM-A87 was determined using a combined strategy of Sanger sequencing and 454 pyrosequencing. About 100 megabases of data were obtained from one 454 (GS-FLX) sequencing run. The sequences were assembled into 144 large contigs that were oriented by Sanger sequencing reads from paired ends of plasmid and fosmid libraries with insert sizes varying from 3 kb and 5 kb to 40 kb. The gaps were closed by primer walking and PCR segment sequencing. The phred-phrap-consed package was used for the assembly and finishing [58], and the finished genome was validated further by 10-kb long PCR.

The tRNA genes were predicted by tRNAscan-SE [59]. The rRNA genes were identified by BLAST search against Rfam [60]. The open reading frames (ORFs) were found by using GLIMMER 3.0 [61]. The predicted ORFs were annotated by similarity searches against databases of nonredundant protein sequences from NCBI, SWISSPROT, Pfam [62], COG [63], KEGG and InterPro [64]. The annotation of ORFs was manually curated with Artemis [65]. Transmembrane regions of the predicted proteins were predicted with TMHMM 2.0 http://www.cbs.dtu.dk/services/TMHMM/. Signal peptide prediction was done with SignalP 3.0 [66]. Clustered regularly interspaced short palindromic repeats (CRISPR) were found with CRISPR-finder http://crispr.u-psud.fr/Server/CRISPRfinder.php. The sequence alignment was done with Clustal X [67].

The compared genome sequences were obtained from the NCBI database FTP site: ftp://ftp.ncbi.nih.gov/genomes/Bacteria/. Amino acid composition and protein isoelectric points were predicted by the EMBOSS Pepstats program. The compared protein numbers were counted by searching the genome annotation file using the protein name with no further refinement of the annotation. COG functional categories were assigned by using a blastp program to search the COG database with all SM-A87 proteins, and the final results were compiled using custom-made Perl scripts.

The complete genome sequence of strain SM-A87 was deposited in GenBank under the accession no. CP001650.

## Authors' contributions

XZ coordinated the study. YZ, BZ and JY designed the project; HD provied the strain; QQ prepared the DNA; XW and GL carried out the sequencing and assembly; QQ and XZ finished the genome; QQ, GL, BX and XZ analyzed the data; QQ wrote the paper; YZ and XC critically reviewed the paper. All authors approved the final manuscript.

## Supplementary Material

Additional file 1 **Operons of TonB-dependent receptor and SusD/RagB family protein**. Predicted operons of TonB-dependent receptor and SusD/RagB family protein as well as adjacent proteins in *Z. profunda *SM-A87 genome. Click here for file

Additional file 2 **Sequence alignment of ZPR_0565 and ZPR_1126 with other initial glycosyltransferases**. CpsE (CAC18355), EpsE (AAC44012), EpsT (EF362569), ExoY (Q02731), GumD (AAA86372), RfbP (P26406), WbaP (AAD21565), WchA (AAK20699). The boxed sequences are conserved amino acid motif of initial glycosyltransferase. Click here for file

Additional file 3 **Polysaccharide biosynthesis clusters**. Two predicted polysaccharide biosynthesis clusters in *Z. profunda *SM-A87 genome. Click here for file

Additional file 4 **Summary of the carbohydrate-degrading enzymes from *Z. profunda *SM-A87**. ^1 ^Y, with signal peptide. ^2 ^aa, amino acids. Click here for file
