# Supplementary material for: The complete genome of Zunongwangia profunda SM-A87 reveals its adaptation to the deep-sea environment and ecological role in sedimentary organic nitrogen degradation
Source: BMC Genomics. 2010 Apr 17;11:247. doi: 10.1186/1471-2164-11-247 (PMC2864250; doi:10.1186/1471-2164-11-247)
Supplement: Additional file 1 — Operons of TonB-dependent receptor and SusD/RagB family protein. Predicted operons of TonB-dependent receptor and SusD/RagB family protein as well as adjacent proteins in Z. profunda SM-A87 genome. [file 1471-2164-11-247-S1.PDF]

Locus_tag	Start position	End position	Strand	Annotation
ZPR_0527	550656	553592	+	TonB-dependent outer membrane receptor
ZPR_0528	553570	555000	+	SusD/RagB family protein
ZPR_0529	555114	557867	+	peptidase M16
ZPR_1020	1110302	1113334	+	TonB-dependent receptor, plug
ZPR_1021	1113346	1114812	+	RagB/SusD domain-containing protein
ZPR_1022	1115221	1117683	+	six-hairpin glycosidase
ZPR_1023	1117721	1120288	+	beta-glucosidase
ZPR_1024	1120290	1122980	+	putative esterase
ZPR_1025	1123000	1124100	+	predicted xylanase
ZPR_1026	1124106	1125278	+	putative esterase
ZPR_1027	1125374	1127080	+	glycosyl hydrolase
ZPR_1028	1127101	1128414	+	glycosyl hydrolase
ZPR_1029	1128453	1130360	+	glycoside hydrolase
ZPR_1030	1130392	1131729	+	glycosyl hydrolase
ZPR_1031	1131745	1133679	+	glycoside hydrolase
ZPR_1032	1133863	1136256	+	putative glycosyl hydrolase
ZPR_1033	1136417	1137934	+	glycosyl hydrolase
ZPR_1282	1394346	1391536	-	protein containing peptidase M16 domain
ZPR_1283	1395750	1394362	-	SusD/RagB family protein
ZPR_1284	1398766	1395761	-	TonB-dependent outer membrane receptor
ZPR_2238	2390423	2387607	-	PqqL-like family 16 peptidase
ZPR_2239	2391798	2390437	-	SusD/RagB family protein
ZPR_2240	2394776	2391849	-	TonB-dependent outer membrane receptor

ZPR_2488	2672789	2675893	+	TonB-dependent receptor, plug
ZPR_2489	2675904	2677481	+	RagB/SusD domain-containing protein
ZPR_2490	2677505	2678347	+	conserved hypothetical protein
ZPR_2491	2678396	2680897	+	glycoside hydrolase family protein
ZPR_2492	2681122	2683533	+	glycoside hydrolase family protein
ZPR_2493	2683615	2685621	+	glycoside hydrolase family 97
ZPR_2935	3172092	3174458	+	glycosyl hydrolases family 31
ZPR_2936	3174890	3177847	+	tonB-dependent receptor plug domain
ZPR_2937	3177866	3179461	+	ragB/SusD family protein
ZPR_3264	3551139	3548431	-	peptidase M16
ZPR_3265	3552634	3551228	-	SusD/RagB family protein
ZPR_3266	3555601	3552641	-	TonB-dependent outer membrane receptor
ZPR_3443	3705718	3708663	+	TonB-dependent outer membrane receptor
ZPR_3444	3708722	3710065	+	SusD/RagB family protein
ZPR_3445	3710178	3712883	+	PqqL-like family 16 peptidase
ZPR_4093	4372857	4375811	+	TonB-dependent outer membrane receptor
ZPR_4094	4375823	4377229	+	SusD/RagB family protein
ZPR_4095	4377240	4380092	+	protein containing peptidase M16 domain
ZPR_4380	4713284	4712109	-	glycosyl hydrolase family 76
ZPR_4381	4714560	4713322	-	conserved hypothetical protein
ZPR_4382	4716095	4714560	-	ragB/SusD family protein
ZPR_4383	4719145	4716140	-	TonB-dependent Receptor Plug Domain
ZPR_4384	4721524	4719206	-	glycosyl hydrolase family 92

ZPR_4647	5025482	5023533	-	alpha-glucosidase
ZPR_4648	5026457	5025540	-	endo-arabinase
ZPR_4649	5027937	5026582	-	putative xylanase
ZPR_4650	5028733	5028101	-	conserved hypothetical protein
ZPR_4651	5030663	5028804	-	ragB/SusD family protein
ZPR_4652	5033844	5030686	-	tonB-dependent Receptor Plug Domain
ZPR_4661	5050180	5048864	-	RagB/SusD domain-containing protein
ZPR_4662	5053465	5050202	-	TonB-dependent receptor, plug
ZPR_4663	5056063	5053499	-	metalloprotease
