# Supplementary figures and images for: The complete genome of Zunongwangia profunda SM-A87 reveals its adaptation to the deep-sea environment and ecological role in sedimentary organic nitrogen degradation
Source: BMC Genomics. 2010 Apr 17;11:247. doi: 10.1186/1471-2164-11-247 (PMC2864250; doi:10.1186/1471-2164-11-247)

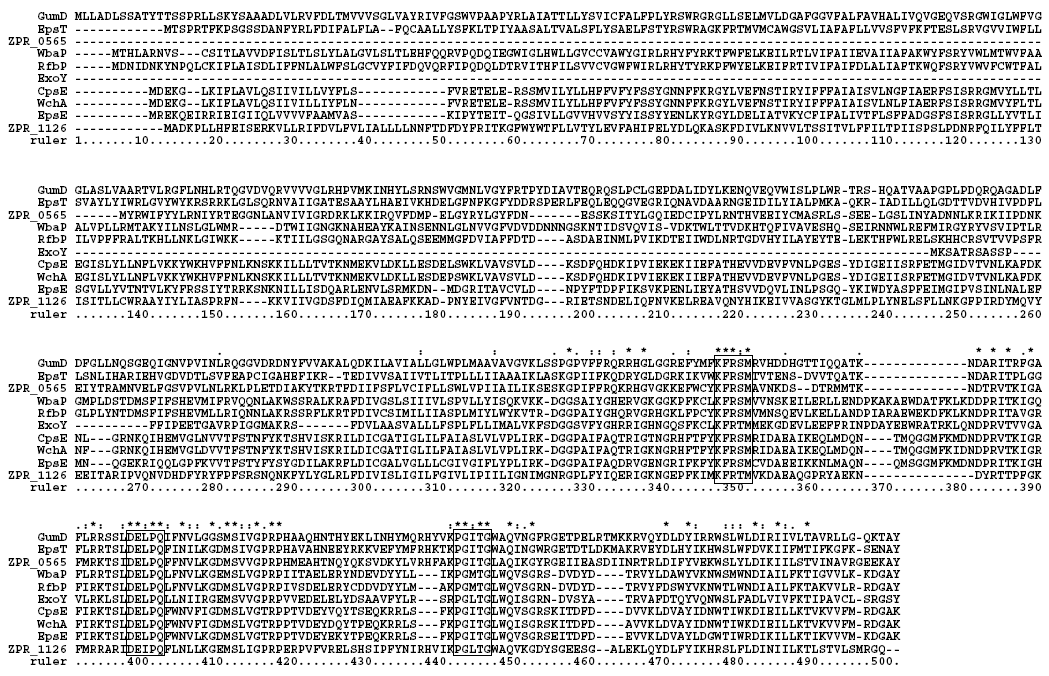

Supplement: Additional file 2 — Sequence alignment of ZPR_0565 and ZPR_1126 with other initial glycosyltransferases. CpsE (CAC18355), EpsE (AAC44012), EpsT (EF362569), ExoY (Q02731), GumD (AAA86372), RfbP (P26406), WbaP (AAD21565), WchA (AAK20699). The boxed sequences are conserved amino acid motif of initial glycosyltransferase. [file 1471-2164-11-247-S2.TIFF]
