# Supplementary material for: The complete genome of Zunongwangia profunda SM-A87 reveals its adaptation to the deep-sea environment and ecological role in sedimentary organic nitrogen degradation
Source: BMC Genomics. 2010 Apr 17;11:247. doi: 10.1186/1471-2164-11-247 (PMC2864250; doi:10.1186/1471-2164-11-247)
Supplement: Additional file 3 — Polysaccharide biosynthesis clusters. Two predicted polysaccharide biosynthesis clusters in Z. profunda SM-A87 genome. [file 1471-2164-11-247-S3.PDF]

Locus_tag	Start position	End position	Strand	Annotation
**Cluster I**				
ZPR_0543	568947	570341	+	UDP-glucose 6-dehydrogenase
ZPR_0544	570403	571353	+	GDP-fucose synthetase
ZPR_0545	571493	572617	+	GDP-D-mannose dehydratase
ZPR_0546	572755	574038	+	polysaccharide biosynthesis protein
ZPR_0547	574082	575050	+	conserved hypothetical protein
ZPR_0548	575047	575622	+	galactoside acetyltransferase (lacA)
ZPR_0549	575619	576734	+	hypothetical protein
ZPR_0550	576737	577330	+	acetyltransferase, CysE/LacA/LpxA/NodL family
ZPR_0551	577343	578593	+	glycosyl transferase group 1
ZPR_0552	578596	579918	+	membrane protein
ZPR_0553	579911	581044	+	glycosyl transferase group 1
ZPR_0554	581076	581255	+	hypothetical protein
ZPR_0555	581333	581704	+	trimeric LpxA-like enzyme
ZPR_0556	581936	582871	+	glycosyl transferase group 1
ZPR_0557	582875	584077	+	glycosyl transferase
ZPR_0558	584202	585158	+	WfeP
ZPR_0559	585273	586535	+	4Fe-4S ferredoxin iron-sulfur binding protein
ZPR_0560	586535	587086	+	transferase hexapeptide repeat containing protein
ZPR_0561	587101	587916	+	glycosyl transferase, group 2 family
ZPR_0562	587913	588851	+	putative dNTP-hexose dehydratase-epimerase
ZPR_0563	588885	589631	+	glycosyl transferase
ZPR_0564	589801	589685	-	hypothetical protein
ZPR_0565	589960	590979	+	glycosyltransferase
ZPR_0566	590980	591747	+	polysaccharide export protein
**Cluster II**				
ZPR_1091	1197555	1197555	+	polysaccharide export outer membrane protein
ZPR_1092	1198323	1198323	+	Ptk-like tyrosine-protein kinase
ZPR_1093	1200858	1200858	+	S23 ribosomal protein
ZPR_1094	1201240	1201240	+	O-antigen export system permease protein
ZPR_1095	1202091	1202091	+	ABC transporter, ATP-binding protein
ZPR_1096	1203334	1203334	+	UDP-Glycosyltransferase/glycogen phosphorylase
ZPR_1097	1204329	1204329	+	glycosyl transferase, family 2
ZPR_1098	1205315	1205315	+	glycosyl transferase family 2
ZPR_1099	1206250	1206250	+	glycosyl transferase family 2
ZPR_1100	1207118	1207118	+	glycosyltransferase
ZPR_1101	1208091	1208091	+	putative UDP-N-acetylglucosamine 2-epimerase
ZPR_1102	1209139	1209139	+	UDP-Glycosyltransferase/glycogen phosphorylase
ZPR_1103	1210575	1210575	+	glycosyl transferase family 2
ZPR_1104	1211352	1211352	+	glycosyl transferase family 2

ZPR_1105	1212439	1212439	+	Glycosyl transferase, group 1
ZPR_1106	1213544	1213544	+	glycosyl transferase
ZPR_1107	1214498	1214498	+	exoV-like protein
ZPR_1108	1216417	1216417	-	integrase
ZPR_1109	1216812	1216812	-	IS3/IS911 family transposase
ZPR_1110	1218165	1218165	-	glycosyl transferase, group 1
ZPR_1111	1219551	1219551	-	UDP-Glycosyltransferase/glycogen phosphorylase
ZPR_1112	1221271	1221271	-	asparagine synthetase
ZPR_1113	1221960	1221960	-	N-acylneuraminate cytidylyltransferase
ZPR_1114	1222742	1222742	-	formyl transferase
ZPR_1115	1223688	1223688	-	polysaccharide deacetylase
ZPR_1116	1224844	1224844	-	UDP-N-acetylglucosamine 2-epimerase
ZPR_1117	1225850	1225850	-	N-acetylneuraminate synthase
ZPR_1118	1226090	1226090	+	glycosyl transferase family protein
ZPR_1119	1227618	1227618	+	glycosyl transferase family protein
ZPR_1120	1228613	1228613	+	Serine acetyltransferase-like protein
ZPR_1121	1229435	1229435	+	glycosyl transferase, group 1
ZPR_1122	1230259	1230259	+	putative glycosyl transferase
ZPR_1123	1231388	1231388	+	O-Antigen Polymerase
ZPR_1124	1232733	1232733	+	glycosyl transferases group 1
ZPR_1125	1233847	1233847	+	Glycosyl transferase, group 1
ZPR_1126	1234705	1234705	+	sugar transferase
