# Supplementary material for: The complete genome of Zunongwangia profunda SM-A87 reveals its adaptation to the deep-sea environment and ecological role in sedimentary organic nitrogen degradation
Source: BMC Genomics. 2010 Apr 17;11:247. doi: 10.1186/1471-2164-11-247 (PMC2864250; doi:10.1186/1471-2164-11-247)
Supplement: Additional file 4 — Summary of the carbohydrate-degrading enzymes from Z. profunda SM-A87. 1 Y, with signal peptide. 2 aa, amino acids. [file 1471-2164-11-247-S4.PDF]

Locus_tag	Signal peptide[1]	Size (aa)[2]	Annotation
**Glycoside hydrolase**			
ZPR_0355		294	putative glycosidase
ZPR_0357		377	glycosidase, PH1107-related
ZPR_0445	Y	384	glycosidase, PH1107-related
ZPR_0611		678	six-hairpin glycosidase
ZPR_0753	Y	564	family 10 glycosyl hydrolase
ZPR_0910		411	glycosyl hydrolase
ZPR_1006		819	six-hairpin glycosidase
ZPR_1013		470	glycoside hydrolase family protein
ZPR_1022		820	six-hairpin glycosidase
ZPR_1027		568	glycosyl hydrolase
ZPR_1028	Y	437	glycosyl hydrolase
ZPR_1029	Y	635	glycoside hydrolase
ZPR_1030		445	glycosyl hydrolase
ZPR_1031	Y	644	glycoside hydrolase
ZPR_1032	Y	797	putative glycosyl hydrolase
ZPR_1033		505	glycosyl hydrolase
ZPR_1048		513	glycosidase
ZPR_1249		741	glycosyl hydrolase family 92
ZPR_1541		1174	glycosyl hydrolases family 2
ZPR_1563		692	six-hairpin glycosidase-like protein
ZPR_1697		128	glycoside hydrolase 15-related protein
ZPR_1698		451	glycoside hydrolase 15-related protein
ZPR_1987	Y	351	glycosyl hydrolases family 43
ZPR_2025		363	BNR repeat-containing glycosyl hydrolase
ZPR_2142	Y	794	putative glycosyl hydrolase
ZPR_2144	Y	333	glycosyl hydrolases family 43

ZPR_2158		473	six-hairpin glycosidase-like protein
ZPR_2159		708	glycosyl hydrolase family 92
ZPR_2491		833	glycoside hydrolase family protein
ZPR_2492	Y	803	glycoside hydrolase family protein
ZPR_2493	Y	668	glycoside hydrolase 97
ZPR_2497		865	glycoside hydrolase family protein
ZPR_2627	Y	471	glycoside hydrolase
ZPR_2818		766	glycoside hydrolase family protein
ZPR_2935		788	glycosyl hydrolases family 31
ZPR_2938	Y	617	glycoside hydrolase family protein
ZPR_3475		397	glycosidase, PH1107-related
ZPR_3478		531	glycoside hydrolase, catalytic core
ZPR_3502		852	glycoside hydrolase family protein
ZPR_3503	Y	764	glycoside hydrolase family protein
ZPR_4166		883	glycosyl hydrolase
ZPR_4177	Y	746	glycosyl hydrolase family 92
ZPR_4179	Y	748	glycoside hydrolase family 92
ZPR_4182		321	glycosidase, PH1107-related
ZPR_4314		546	glycoside hydrolase family 20
ZPR_4333		824	glycoside hydrolase family 2
ZPR_4334		353	glycoside hydrolase, family 5
ZPR_4337		723	putative glycosyl hydrolase
ZPR_4380		391	glycosyl hydrolase family 76
ZPR_4392	Y	482	glycoside hydrolase family 30
**Glucosidase**			
ZPR_0763		800	alpha-glucosidase
ZPR_1023		855	beta-glucosidase
ZPR_1604	Y	782	beta-glucosidase

ZPR_2651		472	beta -glucosidase
ZPR_2817	Y	703	alpha-glucosidase SusB
ZPR_2875	Y	652	putative alpha-glucosidase
ZPR_3036	Y	735	beta-glucosidase
ZPR_3690		748	beta-glucosidase
ZPR_4173	Y	796	putative beta-glucosidase
ZPR_4395	Y	766	beta-glucosidase
ZPR_4647	Y	649	alpha-glucosidase
**Xylanase**			
ZPR_0917	Y	338	endo-1,4-beta-xylanase D precursor
ZPR_0918	Y	325	endo-1,4-beta-xylanase D precursor
ZPR_1025		366	predicted xylanase
ZPR_3039		374	endo-beta-1,4-xylanase
ZPR_3723	Y	303	putative xylanase
ZPR_4649		451	putative xylanase
**Xylosidase**			
ZPR_0915		704	putative beta-xylosidase
ZPR_2730	Y	319	beta-xylosidase
ZPR_4332	Y	337	putative beta-xylosidase
**Beta-galactosidase**			
ZPR_0911	Y	1055	beta-galactosidase
ZPR_1008	Y	1033	beta-galactosidase
ZPR_1388		1029	beta-galactosidase
ZPR_2513	Y	607	probable beta-galactosidase
ZPR_4170	Y	616	putative exported beta-galactosidase

**Alpha-amylase**			
ZPR_2815	Y	481	alpha-amylase
ZPR_2816	Y	615	alpha-amylase
ZPR_2825	Y	481	alpha-amylase precursor
**Chitinase**			
ZPR_1702		214	family 18 chitinase
ZPR_1703	Y	130	chitinase A1 precursor
